# A Novel Method for Speech Acquisition and Enhancement by 94 GHz Millimeter-Wave Sensor

**DOI:** 10.3390/s16010050

**Published:** 2015-12-31

**Authors:** Fuming Chen, Sheng Li, Chuantao Li, Miao Liu, Zhao Li, Huijun Xue, Xijing Jing, Jianqi Wang

**Affiliations:** 1 Department of Biomedical Engineering, Fourth Military Medical University, Xi’an 710032, China; cfm5762@126.com (F.C.); lichuantao614@126.com (C.L.); lium90@163.com (M.L); lizhaofmmu@fmmu.edu.cn (Z.L.); xinyin20130419@163.com (H.X.); fmmujxj@fmmu.edu.cn (X.J.); 2 College of Control Engineering, Xijing University, Xi’an 710123, China; shengli@fmmu.edu.cn; 3 Shaanxi University of Technology, Hanzhong 723001, China

**Keywords:** radar speech, 94 GHz MMW, speech enhancement, empirical mode decomposition, mutual information entropy

## Abstract

In order to improve the speech acquisition ability of a non-contact method, a 94 GHz millimeter wave (MMW) radar sensor was employed to detect speech signals. This novel non-contact speech acquisition method was shown to have high directional sensitivity, and to be immune to strong acoustical disturbance. However, MMW radar speech is often degraded by combined sources of noise, which mainly include harmonic, electrical circuit and channel noise. In this paper, an algorithm combining empirical mode decomposition (EMD) and mutual information entropy (MIE) was proposed for enhancing the perceptibility and intelligibility of radar speech. Firstly, the radar speech signal was adaptively decomposed into oscillatory components called intrinsic mode functions (IMFs) by EMD. Secondly, MIE was used to determine the number of reconstructive components, and then an adaptive threshold was employed to remove the noise from the radar speech. The experimental results show that human speech can be effectively acquired by a 94 GHz MMW radar sensor when the detection distance is 20 m. Moreover, the noise of the radar speech is greatly suppressed and the speech sounds become more pleasant to human listeners after being enhanced by the proposed algorithm, suggesting that this novel speech acquisition and enhancement method will provide a promising alternative for various applications associated with speech detection.

## 1. Introduction

Speech is one of the most important and effective means for human communication, thus, speech acquisition is particularly important. There are some methods which can be used to acquire speech signals, such as traditional air-borne microphones and non-air-borne contact detection. However, traditional microphones are easily disturbed by background noise and their propagation distance is very short, while other methods using non-air-borne contact detection such as electroglottography and the bone conduction microphone constrain people’s free movement and make users feel uncomfortable.

Thus, non-contact speech detection methods have been studied and developed. Optical speech detection technology, as one such approach, had been used to listen for messages. For example, Avargel *et al.* presented a remote speech-measurement system that utilizes an auxiliary laser Doppler vibrometer sensor, and proposed a speech enhancement algorithm to enhance speech quality [1]. Recently, radar sensor speech detection technology has also been investigated by many researchers. In 1998, Holzrichter’s group developed a micro-power impulse radar which was used to measure the movement of the vocal organs [2]. In order to improve the performance synthetic speech and speech pathology as well as allow silent speech recognition, Eid *et al.* explored a novel application of Ultra Wide Band (UWB) radar speech sensing [3]. Chang’s group presented a Doppler radar system and successfully extracted speech information from the vocal vibration signals of a human subject [4]. Although these results verified the effectiveness of the radar sensor in speech, they mainly concentrated on measuring the vibration of the speech organs, instead of examining the performance of the radar speech detection.

Millimeter wave (MMW) radars were developed in previous research for speech detection. Li’s group used MMW radar to detect speech signals, which were successfully acquired with a 40 GHz MMW radar. He also demonstrated that the 60 GHz or 90 GHz radars performed better than the 40 GHz one in this new application [5]. In addition, a MMW radar was examined in our laboratory [6,7]. Li *et al.* successfully used a 34 GHz MMW radar to acquire speech signals in free space [8,9], however, the quality of the 34 GHz MMW radar speech was found to be unsatisfactory. In our previous research, we found that the high operation frequency demonstrated excellent sensitivity for the acquisition of speech signals [10,11,12]. Compared with the Ka-band range, MMW frequency in the W-band range (75–110 GHz) provides a good tradeoff between range and sensitivity for the detection of biosignals [12,13,14].

To further improve sensitivity and achieve high quality speech detection, in this paper a 94 GHz microwave radar sensor with a superheterodyne receiver was employed to acquire speech signals. In addition, in order to avoid the null point, in-phase and quadrature demodulation technology was adopted in this radar. A superheterodyne receiver was employed to reduce the DC offsets and 1/f noise. However, the combined sources of noise, which include ambient, harmonic and electrical circuit noise, were combined in the acquired speech signals. These types of noise greatly degrade the quality of radar speech, and seriously affect the applications of the MMW radar speech. Therefore, how to enhance the quality of radar speech is an important question in radar speech acquisition. Many noise reduction methods have been proposed for enhancing the quality of traditional microphone speech; these include mainly the spectral subtraction, Wiener filtering and wavelet shrinkage methods. However, these methods have several shortcomings which limit their further development. The spectral subtraction method [15] can reduce global noise in speech, but introduces some musical noise. The Wiener filtering method is a linear method which is easy to implement and design [16], but since speech signals are always nonlinear, this results in severe speech distortion. The wavelet shrinkage method relies on the threshold of the wavelet coefficient, and has been applied to denoise signals [17,18]. The application of this method is limited because the basis functions of the algorithm are fixed, and it will not entirely fit real signals. Therefore, it is important for the development of speech enhancement systems to find an adaptive method aimed at improving intelligibility and reducing speech distortion. 

Recently, empirical mode decomposition (EMD) has been proposed by Huang *et al.* for analyzing signals from nonlinear and nonstationary processes [19]. Unlike other nonlinear methods, the basis functions in this case are derived from the signal itself, so the major advantage of the EMD algorithm is its adaptability. Several authors have studied EMD-based signal noise filtering and successfully reduced the noise of signals [20,21,22]. Boudraa *et al.* introduced a new signal denoising approach based on the EMD framework. The approach assumes that the noise of the signal is spread across the intrinsic mode functions (IMFs), and it sets a threshold to remove the noise of the signal; the results show that the EMD-soft method can effectively reduce the signal noise [23]. However, for radar speech, the method should also ensure the intelligibility of the speech when reducing noise. If each IMF is filtered, we find that the noise is suppressed, but the intelligibility of the radar speech is poor. In order to find the best tradeoff between the intelligibility of radar speech and noise reduction, an algorithm combining empirical mode decomposition (EMD) and mutual information entropy (MIE) is proposed for enhancing the perceptibility and intelligibility of radar speech. Mutual information entropy (MIE) is a measure of independence between two variables, a theory proposed by Shannon [24]. In this paper, MIE is used to determine the number of reconstructive components. 

This paper demonstrates a potential radar sensor for acquiring high quality speech, and we find that the quality of the acquired speech was enhanced by our proposed method. The radar sensor can therefore be used for non-contact speech signal detection over long distances. This will provide a promising alternative for various applications associated with speech detection.

## 2. The 94 GHz MMW Radar Sensor

### 2.1. Quadrature Doppler Radar Theory

The 94 GHz MMW radar system typically transmits a single-tone signal by the transmitting antenna, and the signal can be described as below:

(1) $$P_{T}(t)=A\cos(2\pi f_{0}t+\theta_{1})$$

where *A* is the oscillation amplitude, and *f*_0_ is the oscillation frequency of the transmitting signal. *θ*_1_ is the initial phase of the oscillator. When the signal is reflected by the human throat with a distance change *x*(*t*), the received signal may be expressed as [4]:

(2) $$P_{R}(t)=KA\cos(2\pi f_{0}t+\theta_{2}-\frac{4\pi x(t)}{\lambda })$$

where *λ*_0_ is the carrier wavelength of the 94-GHz radar sensor, and *x*(*t*) is the time-varying displacement by a target. *K* is the decay factor of the oscillation amplitude. *θ*_2_ is phase modulated by the nominal distance. Then the received signal and local oscillator signal are mixed, and the mixer signal is filtered by a low-pass filtering. Thus, the signal can be expressed as [25,26]:

(3) $$P_{M}(t)=\frac{KA^{2}}{2}\cos(\Delta \theta +\frac{4\pi x(t)}{\lambda_{0}})+N(t)$$

where Δ*θ* is the constant phase shift dependent on the nominal distance to the target. *N*(*t*) is the phase noise and ambient noise. 

It is known that there is a null detection point problem for a single channel radar. This null detection point occurs with a target distance every *λ*/4 from the radar. In order to avoid the null point of the single-channel radar, a quadrature receiver with I/Q channel was designed [27]. The quadrature receiver with local oscillator phases *π*/2 apart, insuring that there is always at least one output not in the null point. The output of the radar quadrature mixer can be expressed as follows [25,27]:

(4) $$W_{I}(t)=A_{I}\cos(\Delta \theta +\frac{4\pi x(t)}{\lambda_{0}})+N_{I}(t)$$

and:

(5) $$W_{Q}(t)=A_{Q}\sin(\frac{4\pi x(t)}{\lambda_{0}}+\Delta \theta )+N_{Q}(t)$$

where, *A_I_* and *A_Q_* are the amplitudes of the quadrature channel I and channel Q, *N_I_* and *N_Q_* are added sources of noise which include ambient noise and electrical-circuit noise for the I-branch and Q-branch. Therefore, if *A_I_* = *A_Q_*, the associated phase *ω*(*t*) can be extracted by the following equation:

(6) $$\omega (t)=\arctan\left[\frac{W_{Q}(t)-N_{Q}(t)}{W_{I}(t)-N_{I}(t)}\right]=\frac{4\pi x(t)}{\lambda_{0}}+\Delta \theta$$

### 2.2. The 94 GHz MMW Radar System

**Figure 1:**
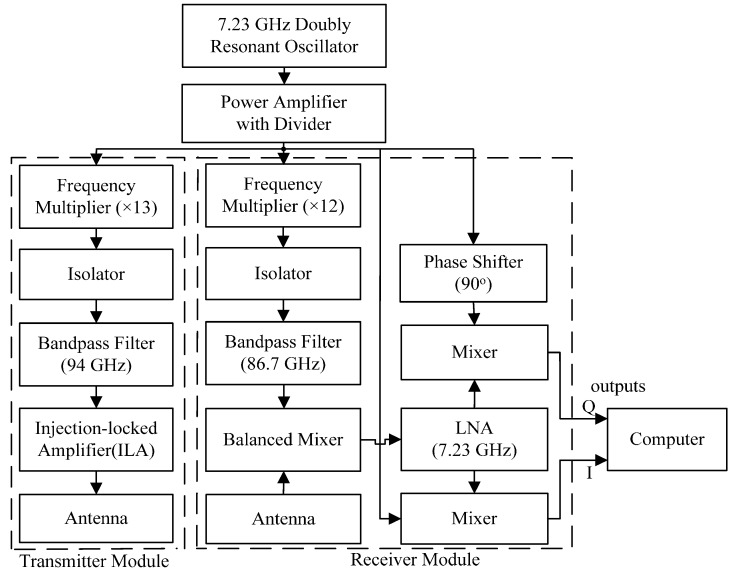
Schematic diagram of the 94 GHz millimeter wave radar sensor.

Figure 1 shows a schematic diagram of the 94 GHz MMW radar sensor system. The system is composed of an oscillator, transmitter module and receiver module. The W-band double resonant oscillator operates at a local frequency at 7.23 GHz and the power of the reference frequency is 20 mW. The transmitting and receiving antennas of the radar sensor are both Cassegrain antennas, with a diameter of 200 mm, a gain of 41.7 dBi, and a beam width of 1° at –3 dB levels. The output radio frequency (RF) power of the transmitting antenna is 100 mW and the equivalent isotropic radiated power (EIRP) is 61.7 dBm. To begin with, the Dielectric Resonator Oscillator (DRO) of 7.23 GHz emits a continuous wave signal, and then the frequency of the signal is amplified and feeds into both the transmitter module and receiver module. In the transmitter module, the local frequency is multiplied 13 times by the frequency multiplier, first it passes through a band-pass filter of 94 GHz, and then generates a high-stability 94 GHz RF signal, with the beams radiated by the transmitting antenna. In the receiver module, the noise figure is 7.6 dB. The total gain of RF-IF is 65 dB and the I/Q phase balance is +/−1 deg. Firstly, the local frequency is multiplied 12 times by the frequency multiplier, and passes through a band-pass filter of 86.7 GHz, and is then balance-mixed with received signal from receiving antenna. Finally, a signal is amplified with a low-noise amplifier (LNA) and is then mixed with two quadrature local signal for the in-phase and quadrature (I/Q) receiver chains. After I/Q quadrature demodulation, the final signal is sampled by an A/D converter to be transferred to a computer, and then the speech signal is recorded by the computer. 

A superheterodyne receiver is employed to avoid the severe DC offsets and the associated 1/f noise at the baseband. Moreover, the transmitting and receiving circuits employ two antennas, and they are separated, which can increase the detection range and reduce interference. The distance and the angle between the two antennas can be easily adjusted. Furthermore, the I/Q quadrature demodulation technology can not only effectively avoid the null detection point problem, but also enhance the signal-to-noise ratio (SNR) by 3 dB compared with the one-signal channel [28].

### 2.3. Safety

To begin with, the safety issue regarding human exposure to radar electromagnetic fields should be taken into account. Thus, the maximum allowed density which exposed to the human should be computed. In this paper, the radiating power of the radar sensor is 100 mW, the antenna gain is 41.7 dBi. The maximum accepted density exposed *S* to the human can be computed as [29]:

(7) $$S\left(\frac{W}{m^{2}}\right)=\frac{\text{raiating}\;\text{power}\times \text{antenna}\;\text{gain}}{4\pi {\left(\text{distance}\right)}^{2}}$$

where the distance represents the minimum distance between the human subject and the radar. Here, the distance is 1 m. Therefore, the maximum acceptable density *S* is about 0.3318 W/m^2^. 

The maximum allowed density level accepted safe power density level of 10 W/m^2^ [30] for human exposure at frequencies from 10 to 300 GHz. The maximum acceptable power density is much lower than the maximum allowed density level accepted safe power density level. Therefore, the radar sensor poses no risk to the human health.

## 3. Experimental Section

### 3.1. Subjects and the Experiment

Ten healthy volunteers (five males and five females) participated in the radar speech experiment. Their ages varied from 20 to 35, and all of them were Chinese native speakers. In the experiment, one of the volunteers sat in front of the radar sensor with his throat kept at the same height as the radar sensor. The radar speech sensor was positioned ranging from 2 m to 20 m away from the subjects. Although the speech signals can be detected at a distance of 20 m, to guarantee high quality speech signals, a distance of 5 m was selected as a representative distance. The volunteers were asked to speak one sentence of Mandarin Chinese “1-2-3-4-5-6”. All of the experimental procedures were in accordance with the rules of the Declaration of Helsinki [31].

### 3.2. Evaluations

In order to test the performance of the proposed algorithm, both objective and subjective methods were applied to assess the results. Signal-noise ratio (SNR), speech spectrogram and mean opinion score (MOS) tests were conducted. In the experiments, three different kinds of background noise—white noise, pink noise and babble noise—were added to the original radar speech. All the types of noise were taken from the NOISEX-92 database, and the noisy radar speech with *SNR_in_* of –5, 0, 5 and 10 dB. In addition, to further illustrate the effectiveness of the proposed algorithm, the results were compared to the spectral subtraction and wavelet shrinkage algorithms. 

The SNR is used as an objective measure to evaluate the proposed method’s performance, and the *SNR_in_* of noisy speech is defined by:

(8) $$SNR_{in}=10\log_{10}\frac{\sum_{n=1}^{N}s^{2}(n)}{\sum_{n=1}^{N}{\left[x(n)-s(n)\right]}^{2}}$$

The *SNR_out_* of the enhanced speech is given by:

(9) $$SNR_{out}=10\log_{10}\frac{\sum_{n=1}^{N}s^{2}(n)}{\sum_{n=1}^{N}{\left[y(n)-s(n)\right]}^{2}}$$

where *x*(*n*) is the noisy speech, *s*(*n*) is the clean speech, *y*(*n*) is the enhanced speech, *N* indicates the number of samples in speech, and *n* represents the sample index.

The speech spectrogram and MOS test are used as the subjective measures to evaluate the proposed method’s performance. From the speech spectrogram, it can be observed that the signal strength of different speech spectra over time, the abscissa of the speech spectrogram represents time, and the ordinate of the speech spectrogram represents frequency. The color depth shows the speech energy value; the deeper the color, the stronger the speech energy. For the MOS test, ten other volunteers were instructed to evaluate the intelligibility of the speech based on the criteria of the mean opinion score test, which is a five point scale (1: bad; 2: poor; 3: common; 4: good; 5: excellent). All listeners were healthy with no reported history of hearing disease. 

## 4. Methods

### 4.1. Empirical Mode Decomposition

**Figure 2:**
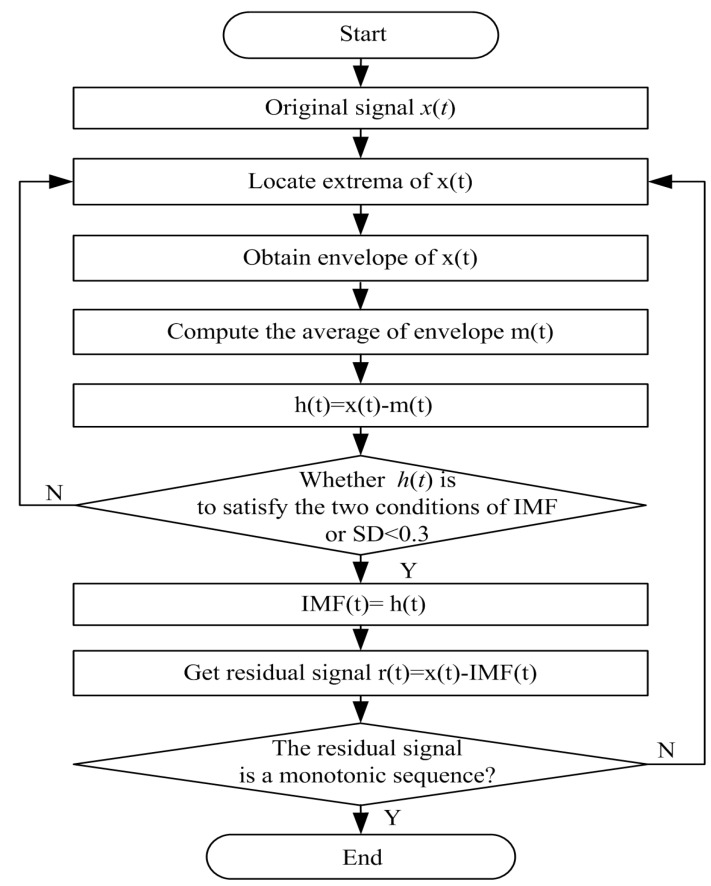
The flow chart of empirical mode decomposition algorithm.

As the core component of the Hilbert Huang transforms (HHT), empirical mode decomposition (EMD) is an adaptive method for processing nonlinear and nonstationary signals [19]. Unlike previous signal processing methods [17,18], the EMD method is intuitive, direct and adaptive. In the whole process of decomposition, all the basis functions are derived from the signal itself. Therefore, the method is very well-suited to processing nonlinear and nonstationary signals [32], such as ECG and speech signal. Given a signal *x*(*t*), EMD can adaptively decompose it into a series of oscillatory components called intrinsic mode functions (IMFs) through the “sifting” process, and each IMF is an oscillatory signal which consists of a subset of frequency components from the original signal. Figure 2 shows the flow chart of the EMD algorithm.

The sifting process can be described as follows:

1. Locate all the extrema (maxima/minima) of *x*(*t*).
2. Interpolate the maxima and minima points by cubic splines to obtain an upper envelope *e_u_*(*t*) and a lower envelope *e_d_*(*t*), respectively.
3. Compute the average *m*_1_(*t*) of the upper and lower envelopes, subtracted from the original signal *x*(*t*) to obtain *h*_1_(*t*) = *x*(*t*) − *m*_1_(*t*).
4. Judging whether *h*_1_(*t*) is to satisfies the following two conditions of IMF:
  (a) In the whole data item, the number of extrema should be equal to the number of zero crossings, or one difference at the most.
  (b) At any point, the mean of the maxima envelope and the minima envelope should be zero. That is to say, signal is symmetric about the time axis.
  If *h*_1_(*t*) satisfies the conditions to be an IMF, it is regarded as the first IMF_1_(*t*), IMF_1_(*t*) = *h*_1_(*t*).
5. If *h*_1_(*t*) does not satisfy the two conditions, the *h*_1_(*t*) is regarded as a new signal, steps 1–4 are repeated on *h*_1_(*t*) to generate the following *h*_2_(*t*). If *h*_2_(*t*) does not satisfy the two conditions, there is a standard deviation (SD) to terminate the sifting process. The stopping criterion is given by:
  (10) $$SD(i)=\sum_{t=0}^{N}\frac{{\left|h_{i-1}(t)-h_{i}(t)\right|}^{2}}{h_{i-1}^{2}(t)}$$
  Usually, the value range of SD is between 0.2 and 0.3 [19]. If *h*_2_(*t*) satisfies the SD, then the IMF_1_(*t*) = *h*_2_(*t*). If *h*_2_(*t*) does not meet the stopping criterion, and the *h*_2_(*t*) is regarded as a new signal, steps 1–5 are repeated on *h*_2_(*t*) to generate the following *h_i_*(*t*), until the *h_i_*(*t*) satisfies the two conditions of IMF or SD. Then, the IMF_1_(*t*) = *h_i_*(*t*).
6. Once the IMF_1_(*t*) is generated and subtracted the original signal to get a residual *r*_1_(*t*): *r*_1_(*t*) = *x*(*t*) − IMF_1_(*t*). The residual signal is treated as the original signal, and steps 1–5 are repeated to get the next residual signal. Therefore, the residual signal can be expressed as *r_n_*(*t*) = *r_n_*_−1_(*t*) − MF*_n_*(*t*). At this point, the *r_n_*(*t*) is a monotonic sequence. After the sifting process, the original signal can be decomposed into several IMF components IMF_1_(*t*), IMF_2_(*t*), … IMF*_n_*(*t*) and a residual sequence *r_n_*(*t*). Therefore, the original signal can be expressed as:
  (11) $$x(t)=\sum_{i=1}^{n}{\mathrm{IMF}}_{i}(t)+r_{n}(t)$$

### 4.2. Mutual Information Entropy

Mutual information entropy is an information theory measurement for quantifying how much information is shared between two or more random variables [33]. It can not only describe the linear correlation between these variables, but also can describe the nonlinear correlation between variables. The major advantage of MIE is that this method can indicate the correlation between two random events without any special requirements for the distribution of the types of variables.

In this paper, MIE is used as a cutoff point to determine the number of reconstructive components. MIE is always non-negative and can measure the relationship between two variables. The MIE *I*(*X*;*Y*) between variables X and Y is defined as [34,35]:

(12) $$I(X;Y)=\sum \sum p(x,y)\log_{2}(\frac{p(x,y)}{p(x)p(y)})$$

Entropy mainly measures the uncertainty of random variables, and the MIE can also be represented by the entropy as:

(13) I(X;Y)=H(X)−H(X|Y)

where:

(14) $$H(X)=-\sum_{x\in \Omega_{x}}p(x)\log_{2}(p(x))$$

and:

(15) $$H(X|Y)=-\sum \sum_{x\in \Omega_{x}}p(x,y)\log_{2}(p(x|y))$$

The more uncertain the event *X* is, the larger *H*(*X*) is. Basically, the stronger the relationship between two variables is, the larger MIE they will have. Zero MIE means the two variables are independent or have no relationship [36].

### 4.3. Selecting the Reconstruction Components 

**Figure 3:**
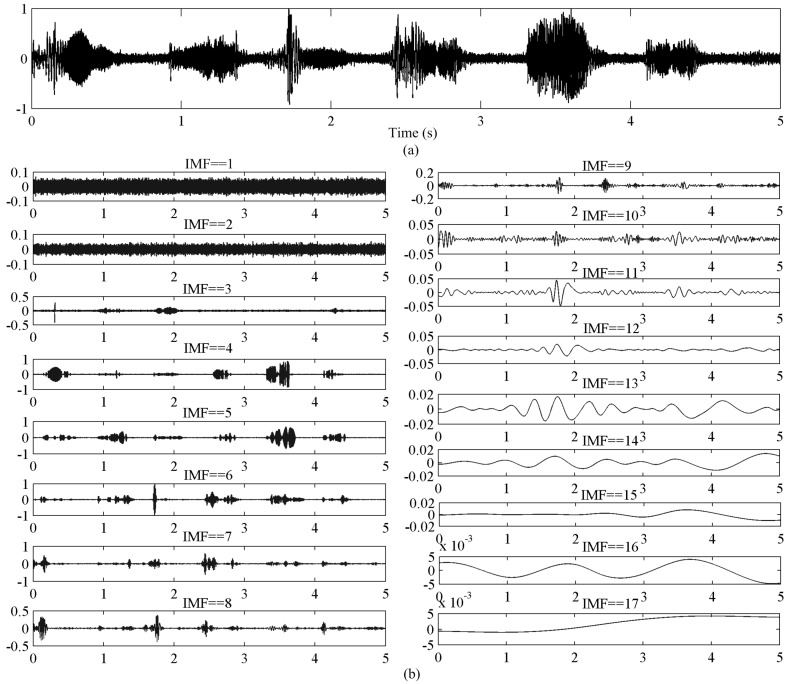
(**a**) The original radar speech signal contaminated by white noise; (**b**) the decomposition of the original radar speech corrupted by white noise using EMD.

Figure 3a shows original radar speech contaminated by white noise. Figure 3b shows the decomposition of the original radar speech signal by EMD. From top to bottom, the frequencies of IMFs decreased gradually. In general, the noise of the signal is spread across the IMFs. From Figure 3b, it is observed that the first three IMFs are mainly noise, and there are few useful original signals. From the fourth to the ninth IMFs, it is observed that there are many useful original signals and the IMFs are very similar to the original signal, but some noise components still remain. From the tenth to the last IMFs, the frequencies of the IMFs are lower and the amplitudes are smaller, and there is detailed information about the original signal. Thus, it is assumed that the original radar speech can be decomposed into high frequency modes, middle frequency modes and low frequency modes. The high frequency modes are mainly noise and interference signal, the middle frequency modes mainly include original useful signals and the low frequency modes mainly are the detailed information from the original signal. In short, the noise is mainly concentrated in the high frequency and middle frequency modes, and there is much less in the low frequency modes.

Some authors have used a wavelet soft-threshold method to remove the noise of IMFs. This method is often employed to process all the IMF components. However, with regard to radar speech, if all the frequency modes are denoised, we find that while the noise is suppressed, the intelligibility of the radar speech is poor. It is because the detailed information from the original signal is removed. Thus, in order to achieve a good tradeoff between radar speech distortion and noise reduction, the high and the middle frequency modes are denoised firstly, and then reconstruct speech signal with the processed IMFs and the remaining low frequency modes.

The mutual information values are sequentially calculated in the adjacent IMF components energy entropy. According to the information theory, the MIE of adjacent IMF components will be in order of large to small, and then back to large:

(16) $$\left\{ \begin{array}{l} \text{If}\;I(IMF_{i},IMF_{i+1})↓\;\text{and}\;I(IMF_{i+1},IMF_{i+2})\;↑\\ k=first(\arg\underset{1\leq i\leq n-1}{\min}[I(IMF_{i},IMF_{i+1})]) \end{array}\right.$$

The point which the minimum MIE appears is selected as the cutoff point to distinguish the high frequency and the middle frequency modes. In order to find the cutoff point of the middle frequency and the low frequency modes, the fixed threshold (FT) was defined as 10^−1^. If the maximum amplitude of IMFs are lower than the FT, it can be assumed that these IMFs are low frequency modes.

### 4.4. The Proposed Algorithm for Radar Speech Enhancement

In the speech enhancement based on the proposed algorithm, the threshold plays an important role in removing noise from radar speech signal. The threshold was estimated by [17,23]:

(17) $$Thr_{i}=\sigma_{i}\sqrt{2\log(N)}$$

where *N* is the signal length, σ is the estimated noise level and is defined by [22]:

(18) $$\sigma =\frac{median\{\left|IMF_{1}(t)-median\{IMF_{1}(t)\}\right|\}}{0.675}$$

In this paper, the soft thresholding function is employed to denoise the high frequency and middle frequency modes for speech enhancement [18,23]:

(19) $$IMF_{i}^{\prime }(t)=\{\begin{array}{cc} sign\{IMF_{i}(t)\}\{IMF_{i}(t)-Thr_{i}\} & |IMF_{i}(t)|\geq Thr_{i}\\ 0 & \left|IMF_{i}(t)\right|\leq Thr_{i} \end{array}$$

Afterwards the high frequency and middle frequency modes are processed by the soft thresholding. Then, the enhanced speech *y*(*t*) is reconstructed with the processed signal $IMF_{i}^{\prime }(t)$ and the remaining low frequency modes. The *y*(*t*) is given by:

(20) $$y(t)=\sum_{i=1}^{k}IMF_{i}^{\prime }(t)+\sum_{k+1}^{n}IMF_{i}(t)$$

where *k* is the number of the high frequency and middle frequency modes, and *n* is the number of IMFs. In conclusion, the proposed algorithm for radar speech enhancement includes the following steps:

1. Decompose the given signal *x*(*t*) into IMFs using the sifting process.
2. Compute the energy entropy of each IMFs using Equations (14) and (15).
3. Compute the MIE of the adjacent IMF components using Equation (13).
4. Determine the cutoff point of high frequency and middle frequency modes using Equation (16).
5. Determine the cutoff point of the middle frequency and low frequency modes using the FT of IMF.
6. Denoise the high frequency and middle frequency modes using Equations (17)–(19).
7. Reconstruct the speech with the processed signal and remaining low frequency modes using Equation (20).

## 5. Results and Discussion

**Figure 4:**
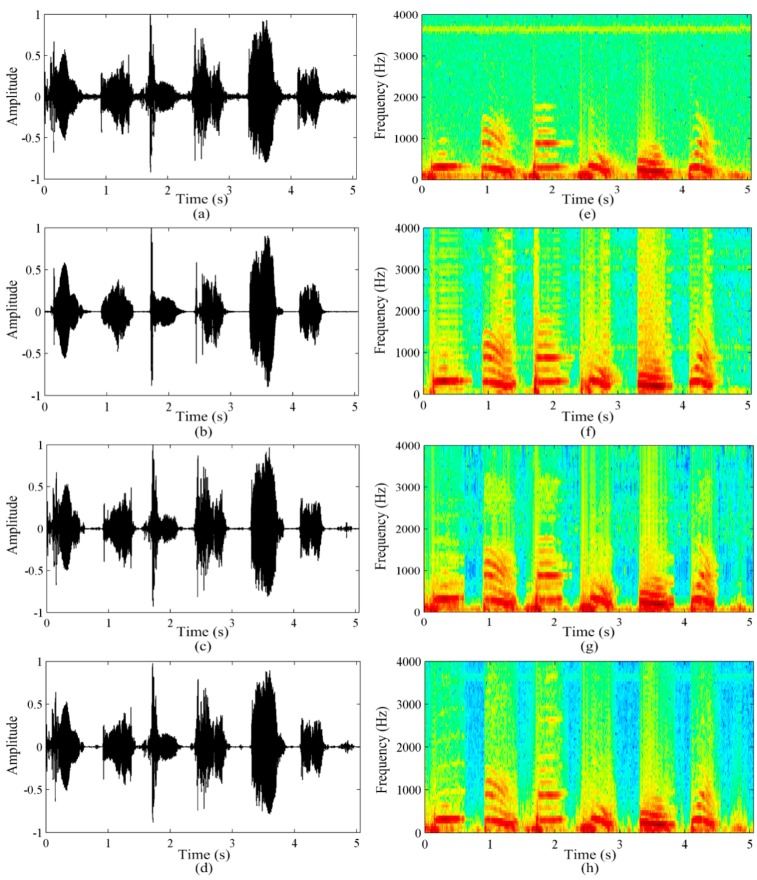
The time-domain waveforms and the spectrograms of the radar speech “1-2-3-4-5-6”. (**a**,**e**) are the original radar speech; (**b**,**f**) are enhanced speech obtained by the spectral subtraction; (**c**,**g**) are enhanced speech obtained by the wavelet shrinkage; (**d**,**h**) are enhanced speech obtained by the proposed algorithm.

This section mainly presents the performance of the proposed algorithm. Speech time domain waveforms and spectrograms are appropriate tools for analyzing speech quality. They can evaluate the extent of noise reduction, residual noise and speech distortion by comparing the original radar speech and the enhanced speech. Figure 4 shows the time-domain waveforms and the spectrograms of the radar speech “1-2-3-4-5-6”. 

Figure 4a,e show the waveform and spectrogram of the original radar speech, respectively. It is observed that the original radar speech signals are contaminated by some noise. Figure 4b–d show the waveforms of the radar speech enhanced by the spectral subtraction algorithm, wavelet shrinkage algorithm and the proposed method, respectively. Figure 4f–h show the corresponding spectrograms of the radar speech enhanced using the three algorithms. Figure 4b,f show that the spectral subtraction algorithm is effective in reducing the combined noise of the radar speech, but the algorithm introduces some new musical noise to the enhanced speech, so the intelligibility of the radar speech was not improved. Figure 4c,g show that the wavelet shrinkage algorithm can also effectively reduce the noise of the radar speech, but in this case the change in the color depth illustrates that the essential information of the speech is removed. This results in severe radar speech distortion. Figure 4d,h show that the proposed EMD and MIE methods not only reduce the low frequency noise in which the combined noise are concentrated, but also eliminates the high frequency noise completely. In addition, to a large extent, the essential signal information of the radar speech is still preserved. These results suggest that the proposed algorithm outperforms the spectral subtraction and wavelet shrinkage algorithms, and that the proposed algorithm is an effective way to improve the quality of radar speech. 

To test the proposed algorithm, a subjective MOS test was used to evaluate the quality of the enhanced radar speech. Ten listeners were selected to listen to the enhanced radar speech sentences using the three algorithms. The results of the averaged MOS under three types of noise at a *SNR_in_* of 5 dB are presented in Table 1. It can be seen from the table that all the scores of the enhanced speech processed by using the three algorithms are improved, especially the proposed method obtained the highest score, between “3” and “4”, followed by the wavelet shrinkage method, with a score of around “3”, meanwhile the spectral subtraction algorithm achieved the lowest score. The results suggest that the proposed method presents the highest speech intelligibility and is more pleasant to the listeners. 

**Table 1:** Comparison of the results of averaged MOS with three types of noise at a SNR of 5 dB. The numbers in the brackets represent standard deviation for these mean opinion scores.

Enhancement Algorithms	White	Pink	Babble
Spectral subtraction	2.78 (0.30)	2.98 (0.38)	2.64 (0.35)
Wavelet shrinkage	3.25 (0.46)	3.37 (0.32)	3.21 (0.27)
Proposed method	3.59 (0.37)	3.71 (0.35)	3.56 (0.42)

The listening tests also indicated the EMD and MIE method is the most suitable for enhancing the radar speech. The method obtained a good tradeoff between the intelligibility and noise reduction. This is because EMD is an adaptive method for processing nonlinear and nonstationary signals, and it does not require presetting fixed basis functions, as all the basis functions are derived from the signal itself. The wavelet shrinkage algorithm will cause severe speech distortion when reducing noise. The spectral subtraction algorithm introduces some musical noise into the enhanced radar speech, so the perceptibility and intelligibility of the radar speech are not improved greatly, and the resulting speech sounds unpleasant to listeners. An objective measurement, the signal-noise ratio, was employed to evaluate the performance of the proposed method. We added babble noise, white noise and pink noise with *SNR_in_* of –5, 0, 5 and 10 dB to the original radar speech. The results of the *SNR_out_* obtained for different noise types and algorithms are seen in Table 2. It can be seen that the three methods lead to an increase of *SNR_out_* values at different *SNR_in_* levels, and the results demonstrate the effectiveness of the three methods. The *SNR_out_* obtained by the proposed method is much higher than those obtained by the spectral subtraction and the wavelet shrinkage algorithms. Even for low *SNR_in_* values, it can be observed the effectiveness of the proposed method in removing the noise components, and we can observe that the spectral subtraction algorithm achieved the worst speech enhancement. Especially at the SNR of 10 dB level, the spectral subtraction led to a decrease of *SNR_out_*. This is due to musical noise being introduced to the speech. The wavelet shrinkage and the proposed algorithm performed better, and this is attributed to the time adaptive threshold strategy. However, the superiority of the proposed method over wavelet shrinkage is due to the adaptive decomposition of the speech signal provided by EMD, as it does not rely on the fixed basis functions.

**Table 2:** Comparison of the SNRs obtained by using three enhancement algorithms.

Enhancement Algorithms	White				Pink				Babble			
	−5	0	5	10	−5	0	5	10	−5	0	5	10
Spectral subtraction	4.1	7.1	8.9	9.7	3.7	6.8	7.4	9.2	2.3	3.7	7.1	8.7
Wavelet shrinkage	4.6	7.6	10.2	12.3	4.1	7.2	8.6	12.1	2.7	5.6	7.3	11.9
Proposed method	5.2	7.5	10.9	14.9	4.8	7.3	10.2	13.7	3.9	6.7	10.1	12.3

## 6. Conclusions

In this paper, a 94 GHz millimeter wave (MMW) radar sensor was employed to acquire speech. A superheterodyne quadrature receiver was designed to reduce the severe DC offsets and the associated 1/*f* noise at the baseband. An EMD and MIE algorithm was designed to enhance radar speech signals, and the performance of proposed algorithm was evaluated by both objective and subjective methods. The results show that human speech can be effectively acquired by a 94 GHz MMW radar sensor when the detection distance is 20 m. The results also show the advantages of the radar speech sensor in long distance detection, preventing acoustic disturbance and ensuring high directivity. Therefore, this novel radar sensor and signal processing method is expected to provide a promising alternative to current methods for various applications associated with speech.
